# Haploidentical transplantation is associated with better overall survival when compared to single cord blood transplantation: an EBMT-Eurocord study of acute leukemia patients conditioned with thiotepa, busulfan, and fludarabine

**DOI:** 10.1186/s13045-018-0655-8

**Published:** 2018-08-30

**Authors:** Federica Giannotti, Myriam Labopin, Roni Shouval, Jaime Sanz, William Arcese, Emanuele Angelucci, Jorge Sierra, Josep-Maria Ribera Santasusana, Stella Santarone, Bruno Benedetto, Alessandro Rambaldi, Riccardo Saccardi, Didier Blaise, Michele Angelo Carella, Vanderson Rocha, Frederic Baron, Mohamad Mohty, Annalisa Ruggeri, Arnon Nagler

**Affiliations:** 1 Department of Hematology and Cell Therapy, Saint-Antoine Hospital, Paris, France; 2 Acute Leukemia Working Party EBMT Paris Office, Hospital Saint- Antoine, Paris, France; 3 Division of Hematology and Bone Marrow Transplantation Division, Chaim Sheba Medical Center, Tel-Hashomer, Sackler School of Medicine, Tel-Aviv University, 52621 Ramat-Gan, Israel; 4 Dr. Pinchas Bornstein Talpiot Medical Leadership Program, Chaim Sheba Medical Center, Tel-Hashomer, Israel; 5 Hospital Universitari y Politecnic La Fe, Valencia, Spain; 6 Instituto Carlos III, CIBERONC, Madrid, Spain; 7 Rome Transplant Network, ¨Tor Vergata¨ University of Rome, Stem Cell Transplant Unit, Policlinico Universitario Tor Vergata, Rome, Italy; 8 Hematology and transplant Unit, Ospedale Policlinico San Martino, Genoa, Italy; 9 Hematology Department, Hospital Santa Creu i Sant Pau, Barcelona, Spain; 10 ICO-Hospital Universitari Germans Trias i Pujol, Josep Carreras Research Institute, Badalona, Spain; 11 Ospedale Civile, Dipartimento di Ematologia, Medicina Trasfusionale e Biotecnologie, Pescara, Italy; 12 S.S.C.V.D Trapianto di Cellule Staminali A.O.U Citta della Salute e della Scienza di Torino, Torino, Italy; 13 Azienda Ospedaliera Papa Giovanni XXIII, Hematology and Bone Marrow Transplant Unit, Bergamo, Italy; 14 Azienda Ospedaliera Universitaria Careggi, Cell Therapy and Transfusion Medicine Unit, Florence, Italy; 15 Programme de Transplantation&Therapie Cellulaire, Centre de Recherche en Cancérologie de Marseille, Institut Paoli Calmettes, Marseille, France; 16 IRCCS, Casa Sollievo della Sofferenza, Department of Hemato-Ooncology, Setm Cell Transplant Unit, San Giovanni Rotondo, Italy; 17 Eurocord, Hôpital Saint Louis, Université Paris-Diderot, Paris, France; 18 Department of Hematology, Churchill Hospital, NHS BT, Oxford University, Oxford, UK; 19 Serviço de Hematologia, Hemoterapia e Terapia Celular, Universidade de São Paulo, São Paulo, SP Brazil; 20 GIGA-I3, University of Liege, Liege, Belgium; 21 Department of Pediatric Hematology and Oncology, IRCCS Bambino Gesù Children’s Hospital, Piazza S Onofrio, 4, 00165 Rome, Italy

**Keywords:** Acute myeloid leukemia, Stem cell transplantation, Conditioning regimens, Thiotepa-busulfan-fludarabine, Haploidentical stem cell transplantation, Umbilical cord blood transplantation

## Abstract

**Background:**

Thiotepa-busulfan-fludarabine (TBF) is a widely used conditioning regimen in single umbilical cord blood transplantation (SUCBT). More recently, it was introduced in the setting of non-T cell depleted haploidentical stem cell transplantation (NTD-Haplo). Whether TBF based conditioning provides additional benefit in transplantation from a particular alternative donor type remains to be established.

**Methods:**

This was a retrospective study based on an international European registry. We compared outcomes of de-novo acute myeloid leukemia patients in complete remission receiving NTD-Haplo (*n* = 186) vs. SUCBT (*n* = 147) following myeloablative conditioning (MAC) with TBF. Median follow-up was 23 months. Treatment groups resembled in baseline characteristics.

**Results:**

SUCBT was associated with delayed engraftment and higher graft failure. In multivariate analysis no statistically significant differences were observed between the two groups in terms of acute or chronic graft-versus-host disease (GvHD) (HR = 1.03, *p* = 0.92 or HR = 1.86, *p* = 0.21) and relapse incidence (HR = 0.8, *p* = 0.65). Non-relapse mortality (NRM) was significantly higher in SUCBT as compared to NTD-Haplo (HR = 2.63, *p* = 0.001); moreover, SUCBT did worse in terms of overall survival (HR = 2.18, *p* = 0.002), leukemia-free survival (HR = 1.94, *p* = 0.007), and GvHD relapse-free survival (HR = 2.38, *p* = 0.0002).

**Conclusions:**

Our results suggest that TBF-MAC might allow for a potent graft-versus-leukemia, regardless of the alternative donor type. Furthermore, in patients receiving TBF-MAC, survival with NTD-Haplo may be better compared to SUCBT due to decreased NRM.

**Electronic supplementary material:**

The online version of this article (10.1186/s13045-018-0655-8) contains supplementary material, which is available to authorized users.

## Background

Allogeneic hematopoietic stem cell transplantation (HSCT) is a potential curative treatment for patients with acute myeloid leukemia (AML) [1]. The introduction of transplantation from alternative donors, i.e., unrelated umbilical cord blood transplantation (UCBT) and haploidentical transplantation (Haplo), has increased the availability of this treatment. UCBT and Haplo are considered a valid option for patients with acute leukemia lacking a human leukocyte antigen (HLA) matched sibling or unrelated donor, or when transplantation cannot be delayed [2–6]. Stem cells from both types of donors are readily available. In the UCBT setting the process of stem cell collection is risk-free to the donor, and the graft is relatively permissive to HLA incompatibility [7–11]. Contemporary transplantation practice involving the use of double cord blood units in case that there are not enough stem cells in a single cord, flexible conditioning regimens, effective graft-versus-host disease (GvHD) prophylaxis platforms with non-T cell depleted (NTD) Haplo, and improved management of post-transplant complications, have brought improvement in outcomes of alternative donor transplantations [3, 7, 12]. Several studies have reported that results with UCBT and Haplo are comparable with those of transplants from HLA identical or matched unrelated donors [13–22].

Conditioning regimens are administered as part of the transplant procedure to prevent graft rejection by immunoablation and in order to reduce the tumor burden. As the graft versus tumor effect was recognized to contribute to the effectiveness of HSCT, reduced-intensity and nonmyeloablative conditioning regimens have been developed, making HSCT applicable to older or unfit patients [23]. Still, myeloablative conditioning (MAC) regimens remain the preferred option in adult patients (age ≤ 55 years) with high-risk acute leukemia [24]. Despite the availability of various effective conditioning protocols, standard regimens have yet to be established for the different types of HSCT in the various malignancies, leading to high heterogeneity in clinical practice [25]. Therefore, characterizing the effects of a specific regimen in a particular disease category is of major clinical importance.

The use of thiotepa–IV busulfan–fludarabine (TBF) at a myeloablative dose in single unit UCBT (SUCBT) was pioneered by the Valencia group, which reported high rates of engraftment and long-term disease-free survival in patients transplant at early disease stage of hematological malignancies [26]. TBF is widely applied in UCBT and its efficacy is well established [27]. Conditioning protocols in the Haplo setting are more heterogeneous and often determined according to institutional policies [2, 28–32]. More recently, TBF has been increasingly employed in Haplo transplantation with favorable outcomes [31, 32]. Comparing the outcome between patients receiving an allogeneic HSCT from alternative donors is an unmet need. Therefore, we retrospectively analyzed and compared the results of allogeneic HSCT with myeloablative TBF-based conditioning, in a homogeneous population of AML adult patients in complete remission (CR) receiving either NTD-Haplo (*n* = 186) or SUCBT (*n* = 147). The analysis was based on data reported to the European Society for Blood and Marrow Transplantation (EBMT) Acute Leukemia Working Party (ALWP), Cellular Therapy and Immunobiology Working Party, and the Eurocord registry.

## Methods

### Study design and definition

We retrospectively analyzed patients aged ≥18 years diagnosed with de novo AML, who received a first HSCT either from an NTD haploidentical-related donor (recipient-donor number of mismatches ≥ 2) (*n* = 186) or an unmanipulated single cord blood unit (*n* = 147). Data were reported by the ALWP of the EBMT and EUROCORD, between January 2007 and December 2015. Minimal HLA typing requirements for UCBT followed the current practice of antigen level typing for HLA-A and -B and allele-level typing of HLA-DRB1. For patients receiving Haplo, peripheral blood or bone marrow was used as a stem cell source, without ex vivo T cell depletion. Transplants were performed in 75 EBMT transplant centers: 17 performed only SUCBT, 44 only Haplo, and 14 centers performed both procedures. All patients were given a myeloablative reduced toxicity conditioning regimen consisting of thiotepa, IV busulfan, and fludarabine. TBF-MAC was defined as a regimen containing a total dose of IV busulfan ≥ 9.6 mg/kg [33]. Cytogenetic risk groups were defined according to the Medical Research Council (MRC) classification system [34].

All patients provided informed consent for transplants according to the Declaration of Helsinki. The Review Boards of the ALWP of EBMT, and Eurocord approved this study.

### Endpoints

The primary endpoint was leukemia-free survival (LFS). LFS was defined as survival without leukemia or relapse following transplantation. GvHD-free relapse-free survival (GRFS) events were defined as grade 3–4 acute GvHD, extensive chronic GvHD, disease relapse, or death from any cause [35]. Overall survival (OS) was calculated from the date of transplant until death from any cause or last observation alive. Relapse incidence (RI) was defined as the occurrence of disease after transplantation, determined by morphological evidence of the disease in bone marrow, blood, or extramedullary organs. Non-relapse mortality (NRM) was defined as death without prior relapse.

Neutrophil recovery was defined as achieving absolute neutrophil count of 0.5 × 10^9^/l for three consecutive days. Acute and chronic GvHD was defined using the standard criteria [36, 37].

### Statistical analysis

Median values and ranges were used for continuous variables and percentages for categorical variables. For each continuous variable, the study population was initially split into quartiles and into two groups by the median. Patient-, disease-, and transplant-related variables of the groups were compared using chi-square or Fischer’s exact test for categorical variables, and the Mann–Whitney test for continuous variables. The probabilities of OS, LFS, and GRFS were calculated using the Kaplan–Meier method and the log-rank test for univariate comparisons [38]. The probabilities of neutrophil engraftment, grade II–IV acute and chronic GvHD, relapse, and NRM were calculated with the cumulative incidence method and Gray test for comparisons. Multivariate analyses adjusted for differences between the groups were performed using the Cox proportional hazards regression model for LFS and OS, and for engraftment, GvHD, NRM, and relapse [39].

The final model was adjusted for the following variables: transplant strategy (Haplo or SUCBT), disease status at HSCT (first or second CR), time from diagnosis to HSCT, age at transplant, year of HSCT, donor/recipient sex match, Karnofsky performance status (KPS), and center effect. *p* values were two-sided. Statistical analyses were performed with the SPSS 22 (SPSS Inc./IBM, Armonk, NY, USA) and R 3.0 (R Development Core Team, Vienna, Austria) software packages.

## Results

### Patients, disease, and transplant characteristics

Patient and disease characteristics are summarized in Table 1. Per protocol, all patients received a TBF-MAC-based regimen. The two populations were overall homogeneous in terms of patients and disease characteristics, except for median age at transplant which was older for NTD-Haplo (44 [range, 19–66] vs. 42 [range, 18–68], *p* = 0.046). Most patients were in first CR (NTD-Haplo, 70% vs. SUCBT, 77% *p* = 0.14); median interval from diagnosis to transplant was also similar (176 vs. 194 days; *p* = 0.09). Cytogenetic risk groups were alike between the Haplo and SUCBT groups (*p* = 0.76), with intermediate risk being most prevalent (36% vs. 41%, respectively). Haplo transplantations were performed in more recent years (median year of transplantation was 2014 vs. 2011; *p* < 0.001). As expected, anti-thymocyte globulin (ATG) was mostly used in SUCBT (91% vs. 29% in NTD-Haplo; *p* < 0.001). For SUCBT, the median dose of total nucleated cells at collection was 3.3 × 10^7^/kg (range, 1.7–8.4), and 80% of the patients received ≥ 2.5 × 10^7^/kg. Cord blood units were HLA matched with the recipient at a level of at least 4/6 in 68% of the cases. Among NTD-Haplo patients, 80% received bone marrow as stem cell source, and post-transplant cyclophosphamide (PTCY) was administrated in 71% of the cases (Additional file 1: Table S1). Further details about transplant procedures and GvHD prophylaxis are provided in (Additional file 1: Tables S2, S3). The median follow-up was 22 (range, 1–96) and 24 (range, 1–83) months for NTD-Haplo and SUCBT, respectively.

**Table 1:** Population characteristics

	NTD-Haplo (*n* = 186)	SUCBT (*n* = 147)	*p* value
Follow-up, months median (range)	22.07 (0–96.3)	24.42 (0–83.1)	
HSCT year, median (range)	2014 (2008–2015)	2011 (2007–2015)	< 0.001
Age, years. median (range)	44.3 (18.5–66.1)	42.6 (18–67.9)	0.046
Recipient sex			
Male	85 (45.7%)	65 (44.2%)	0.787
Female	101 (54.3%)	82 (55.8%)	
Missing	0	6	
Karnofsky performance status			
< 90	21 (12.5%)	15 (15.31%)	0.519
≥ 90	147 (87.5%)	83 (84.69%)	
Missing	18	49	
Interval from diagnosis to HSCT, months, median (range)	6.6 (2.1–189.6)	6 (3–214.2)	0.097
Disease status at HSCT			
CR1	130 (69.9%)	113 (76.9%)	0.154
CR2	56 (30.1%)	34 (23.1%)	
MRC risk classification			
Good	16 (8.6%)	13 (8.8%)	0.762
Intermediate	67 (36.0%)	60 (40.8%)	
Poor	19 (10.2%)	16 (10.9%)	
Missing	84 (45.2%)	58 (39.5%)	
Female donor to male recipient			
No	143 (76.9%)	108 (76.6%)	0.952
Yes	43 (23.1%)	33 (23.4%)	
Recipient CMV serostatus			< 0.001
Negative	35 (19.1%)	29 (25.9%)	
Positive	148 (80.9%)	83 (74.1%)	
Missing	3	35	
In-vivo T cell depletion (ATG)			
No	131 (71.2%)	13 (9.0%)	< 0.001
Yes	53 (28.8%)	131 (91.0%)	
Missing	2	3	

*NTD-Haplo* Non-T cell depleted haploidentical transplantation, *SUCBT* single umbilical cord blood transplantation, *HSCT* hematopoietic stem cell transplantation, *CR* complete remission, *MRC* Medical Research Council, *CMV* cytomegalovirus, *ATG* antithymocyte globulin

### Engraftment

The cumulative incidence of neutrophil engraftment at day 60 after NTD-Haplo and SUCBT was 96% vs. 86% (*p* < 0.001), respectively. The median time for neutrophil recovery was 18 (range − 8-38) days for Haplo and 21 (range 11–57) days for SUCBT, (*p* < 0.001). Twenty patients did not engraft after SUCBT; of these, two are alive at 10 and 62 months, respectively, both after salvage with a second transplant from a haploidentical-related donor. The remaining 18 patients died in a median time of 1 month (range, 0–7), one patient after an autologous back-up. Among the seven patients who did not engraft after NTD-Haplo, none are alive, with a median time to death of 1.74 months (range, 0.3–17.22). Three of these patients received a second allogeneic transplantation, and only one engrafted, surviving more than 1 year.

### Acute and chronic GvHD

The cumulative incidence of day 100 grade II–IV acute GvHD was 26% and 29% after NTD-Haplo and SUCBT (*p* = 0.85), respectively (Table 2). Cumulative incidence of grade III–IV acute GvHD was 7% in both groups (*p* = 0.99). The cumulative incidence of chronic GvHD was 33% after NTD-Haplo and 37% after SUCBT (*p* = 0.49). In the multivariate analysis (Table 3), no significant difference was found between the two groups in terms of acute or chronic GvHD (hazard ratio (HR) = 1.03, *p* = 0.92; HR = 1.86, *p* = 0.92, respectively). A center effect was found for chronic GvHD (*p* < 0.001).

**Table 2:** Univariate analysis

	Acute GvHD II–IV [95% CI]	Acute GvHD III–IV [95% CI]	2-year chronic GvHD [95% CI]	2-year relapse [95% CI]	2-year NRM [95% CI]	2-year LFS [95% CI]	2-years OS [95% CI]	2-year GRFS [95% CI]
Donor type								
NTD-Haplo	25.6% [19.4–32.2]	6.8% [3.7–11.2]	33% [25.2–40.9]	16.6% [11.1–23.1]	20.6% [14.7–27.2]	62.8% [55.1–70.6]	69.2% [61.9–76.6]	55.5% [47.5–63.5]
SUCBT	28.5% [21.3–36]	7% [3.6–12]	37.1% [27–47.2]	11.6% [6.9–17.6]	48.4% [39.4–56.8]	40% [31.5–48.6]	41.7% [33–50.4]	30.3% [22.3–38.3]
*p* value	0.853	0.997	0.492	0.709	< 0.001	< 0.001	< 0.001	< 0.001
HSCT year								
≤ 2013	26.5% [20.6–32.9]	6.2% [3.4–10.2]	37.1% [29.4–44.8]	14.1% [9.6–19.4]	37.4% [30.5–44.3]	48.5% [41.4–55.7]	52.8% [45.7–59.9]	41.1% [34.1–48.1]
> 2013	27.4% [19.8–35.4]	8% [4.1–13.6]	27.6% [18.4–37.6]	14.6% [8.3–22.7]	26.3% [17.4–36]	59.1% [48.5–69.7]	61.4% [50.1–72.8]	49.5% [38.9–60.2]
*p* value	0.780	0.483	0.278	0.834	0.027	0.022	0.023	0.142
Age, years								
< 44	30.7% [23.7–37.9]	6.3% [3.2–10.8]	32.6% [24.1–41.3]	16.5% [10.9–23.1]	28% [20.9–35.5]	55.5% [47.2–63.7]	61% [53–69]	48.2% [39.9–56.6]
≥ 44	23% [16.8–29.8]	7.5% [4.1–12.2]	36.3% [27.4–45.2]	11.8% [7.2–17.8]	38.6%[30.5–46.6]	49.6% [41.2–57.9]	52.2% [43.7–60.7]	39.2% [30.9–47.4]
*p* value	0.133	0.683	0.355	0.576	0.043	0.137	0.089	0.090
Karnofsky performance status								
< 90	31.6% [16.9–47.3]	14.4% [5.1–28.2]	33.4% [14.7–53.4]	20.5% [8.8–35.6]	49.2% [29.9–65.9]	30.2% [13.5–47]	41.1% [23.4–58.8]	22.9% [7.1–38.7]
≥ 90	26.3% [20.7–32.3]	5% [2.6–8.4]	32.2% [25–39.6]	12.9% [8.6–18.1]	29.1% [22.9–35.7]	58% [50.9–65]	59.8% [52.8–66.8]	49.2% [42–56.5]
*p* value	0.634	0.042	0.880	0.085	0.017	< 0.001	0.007	0.002
Interval from diagnosis to HSCT, months								
≤ 6.3	25.2% [18.7–32.2]	6.3% [3.2–10.9]	33.9% [25.3–42.7]	15.9% [10.1–22.8]	33.3% [25.4–41.3]	50.8% [42.2–59.5]	56.4% [47.8–65]	40% [31.4–48.5]
> 6.3	28% [21.3–35.1]	7.5% [4.1–12.3]	34.8% [25.9–43.8]	12.9% [8.2–18.8]	33.4% [26–41]	53.7% [45.7–61.7]	56.7% [48.7–64.7]	47.7% [39.6–55.7]
*p* value	0.502	0.665	0.955	0.718	0.713	0.830	0.676	0.576
Disease status at HSCT								
CR1	26.8% [21.3–32.6]	7.3% [4.4–11.1]	36% [28.8–43.2]	13.9% [9.6–19.1]	33.2% [26.9–39.7]	52.9% [45.9–59.8]	57.7% [50.8–64.5]	42.8% [35.8–49.7]
CR2	26.8% [17.9–36.6]	5.8% [2.1–12.2]	29.8% [18.3–42.2]	15% [8.2–23.9]	33.3% [23.1–43.9]	51.6% [40.4–62.9]	54.1% [42.9–65.2]	46.2% [35.1–57.3]
*p* value	0.965	0.654	0.367	0.591	0.906	0.618	0.376	0.960
Female donor to male recipient								
No	25.7% [20.4–31.4]	5.4% [3–8.8]	32.1% [25.1–39.2]	14% [9.7–19.1]	30.2% [24.2–36.5]	55.8% [49–62.6]	60.2% [53.6–66.9]	46.8% [39.9–53.7]
Yes	31.5% [21.2–42.3]	12.3% [6–21]	42.4% [29–55.2]	13.3% [6.4–22.6]	41.9% [29.8–53.5]	44.8% [32.7–57]	48.5% [36.2–60.8]	36.2% [24.6–47.8]
*p* value	0.295	0.041	0.133	0.485	0.073	0.035	0.055	0.063

*GvHD* Graft-versus-host disease, *NRM* non-relapse mortality, *LFS* leukemia-free survival, *OS* overall survival, *GRFS* GvHD-free relapse-free survival, *CI* confidence interval, *NTD-Haplo* non-T cell depleted Haploidentical transplantation, *SUCBT* single umbilical cord blood transplantation, *HSCT* hematopoietic stem cell transplantation, *CR* complete remission

**Table 3:** Multivariable analysis

	Acute GvHD II–IV		Chronic GvHD		Relapse		NRM		LFS		OS		GRFS	
	HR (95% CI)	*p*	HR (95% CI)	*p*	HR (95% CI)	*p*	HR (95% CI)	*p*	HR (95% CI)	*p*	HR (95%CI)	*p*	HR (95% CI)	*p*
Donor type														
SUCBT vs. Haplo	1.03 (0.56–1.89)	0.923	1.86 (0.70–4.95)	0.21	0.81 (0.32–2.04)	0.65	2.63 (1.44–4.83)	0.002	1.95 (1.20–3.16)	0.007	2.19 (1.31–3.65)	0.003	2.39 (1.51–3.79)	< 0.001
Year of HSCT	0.97 (0.85–1.11)	0.677	0.91 (0.77–1.08)	0.30	0.93 (0.76–1.14)	0.50	1.01 (0.88–1.15)	0.925	0.98 (0.88–1.10)	0.772	1.00 (0.89–1.12)	1.000	1.07 (0.97–1.189)	0.197
Age (per 10 years)	0.89 (0.72–1.08)	0.236	1.17 (0.93–1.47)	0.19	1.07 (0.80–1.42)	0.66	1.20 (0.99–1.47)	0.071	1.15 (0.98–1.35)	0.096	1.18 (0.99–1.40)	0.055	1.11 (0.96–1.29)	0.164
Karnofsky PS														
≥ 90 vs. < 90	0.67 (0.35–1.31)	0.243	1.05 (0.44–2.55)	0.91	0.35 (0.15–0.81)	0.01	0.60 (0.33–1.09)	0.094	0.50 (0.31–0.81)	0.005	0.60 (0.36–1.00)	0.053	0.58 (0.36–0.92)	0.020
Disease status at HSCT														
CR2 vs. CR1	1.16 (0.68–1.98)	0.582	0.89 (0.44–1.79)	0.74	0.79 (0.34–1.84)	0.59	1.19 (0.70–1.20)	0.524	1.07 (0.69–1.66)	0.760	1.19 (0.76–1.87)	0.457	0.92 (0.61–1.39)	0.703
Female donor to male recipient														
Yes vs. No	1.63 (0.97–2.73)	0.064	1.15 (0.60–2.18)	0.67	1.25 (0.57–2.73)	0.58	1.84 (1.12–3.02)	0.016	1.67 (1.11–2.53)	0.014	1.67 (1.08–2.58)	0.020	1.55 (1.05–2.28)	0.027
Center		0.935		< 0.001		0.94		0.262		0.924		0.278		0.206

*GvHD* Graft-versus-host disease, *NRM* non-relapse mortality, *LFS* leukemia-free survival, *OS* overall survival, *GRFS* GvHD-free relapse-free survival, *CI* confidence interval, *SUCBT* single umbilical cord blood transplantation, *NTD-Haplo* non-T cell depleted Haploidentical transplantation, *HSCT* hematopoietic stem cell transplantation, *CR* complete remission

### Relapse and NRM

**Fig. 1:**
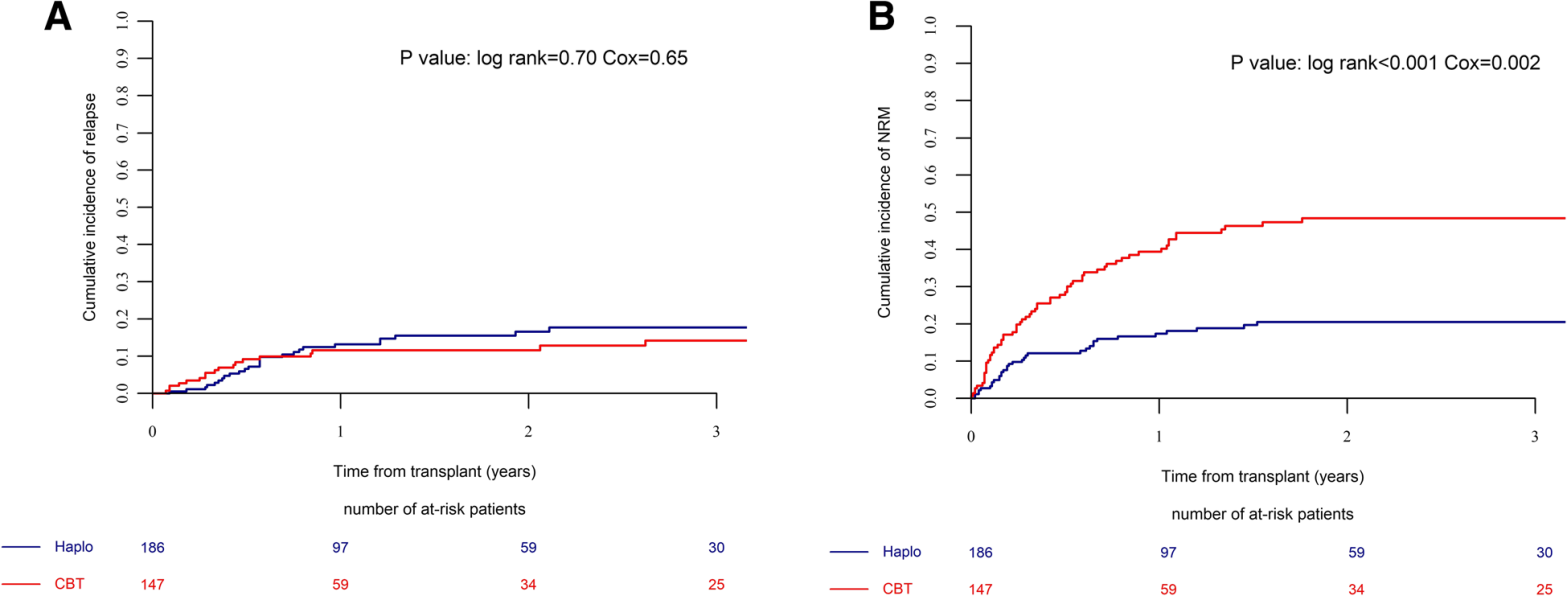
The cumulative incidence of relapse (**a**) and NRM (**b**) by donor type. The numbers of patients at risk for the event are included below each graph. Haploidentical transplantation (Haplo) and single umbilical cord blood transplantation (CBT)

The 2-year RI was 17% for NTD-Haplo vs. 12% for SUCBT (*p* = 0.7) (Fig. 1a, Table 2). In the multivariate analysis (Table 3), relapse was not statistically different between the two groups of patients (HR = 0.8, *p* = 0.65). However, it was lower in patients who had a good KPS (≥ 90) at transplant (HR = 0.35, *p* = 0.01). NRM at 2 years was 21% and 48% for NTD-Haplo and SUCBT (*p* < 0.001), respectively (Fig. 1b, Table 2). The causes of death are listed in Additional file 1: Table S4. The multivariate model confirmed a significantly higher risk of NRM in the SUCBT group (HR = 2.63, *p* = 0.002). Also, NRM was higher in male recipients receiving a female donor (HR 1.84, *p* = 0.015), independently of the stem cell source. Infections and GvHD were the most common causes of transplant-related deaths in both groups (NTD-Haplo vs. SUCBT, infections 35% vs. 45%; GvHD 20% vs. 19%). Disease recurrence accounted for 27% and 16% of deaths after NTD-Haplo and SUCBT, respectively.

### OS, LFS, and GRFS

**Fig. 2:**
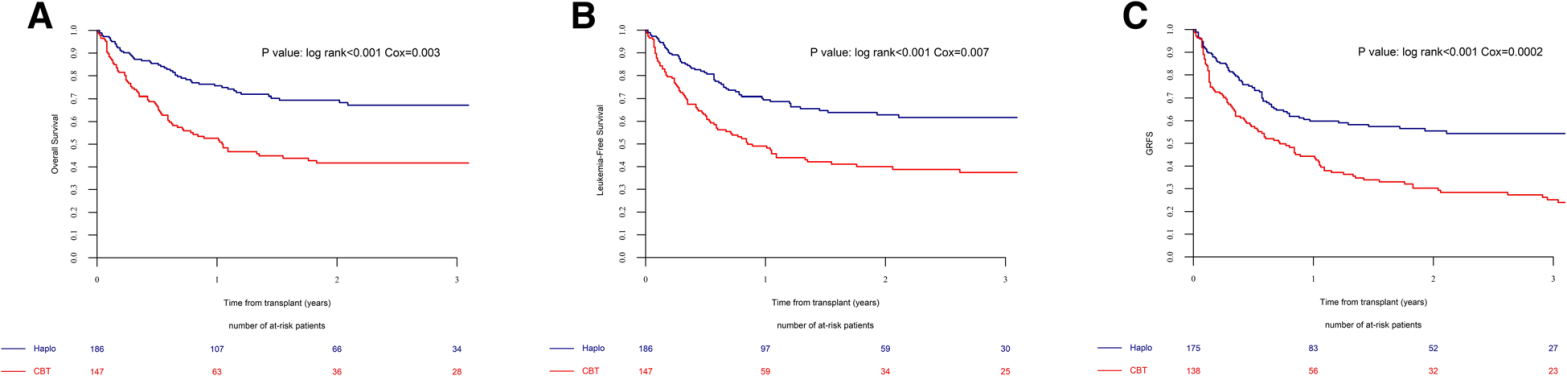
The estimated probability of overall survival (**a**), leukemia-free survival (**b**), and GRFS (**c**) by donor type. The numbers of patients at risk for the event are included below each graph. Graft-versus-host disease-free relapse-free survival (GRFS), haploidentical transplantation (Haplo), and single umbilical cord blood transplantation (CBT)

The probability of 2-year OS, LFS, and GRFS in the NTD-Haplo vs. SUCBT groups were 69% vs. 42% (*p* < 0.001), 63% vs. 40% (*p* < 0.001), and 56% vs. 30% (*p* < 0.001), respectively (Table 2). The benefit of NTD-Haplo was maintained in a sub-analysis restricted to patients with intermediate cytogenetic risk (Additional file 1: Table S5). In the multivariate analysis (Table 3), the type of donor had a statistically significant impact on OS, LFS, and GRFS, which were significantly lower in SUCBT as compared to NTD-Haplo (OS, HR = 2.18, *p* = 0.003; LFS, HR = 1.93, *p* = 0.007; GRFS, HR = 2.38, *p* = 0.0002). The use of female donors for male recipients was independently associated with lower OS, LFS overall survival (HR = 2.18, *p* = 0.002) and GRFS (HR = 1.67, *p* = 0.02; HR = 1.67, *p* = 0.014; and HR = 1.54, *p* = 0.026), while a KPS ≥ 90 at transplant was associated with higher LFS and GRFS (HR = 0.5, *p* = 0.004 and HR = 0.57, *p* = 0.02) Fig. 2.

## Discussion

TBF is a well-established conditioning regimen in SUCBT and has more recently brought into use in Haplo transplantations [26, 27, 31, 32]. In this retrospective analysis, we compare outcomes of NTD-Haplo and SUCBT in a population of AML patients conditioned with TBF at a myeloablative dose. Overall, the treatment groups resembled with regard to baseline characteristics. The risk for relapse and acute and chronic GvHD were similar regardless of donor type. Engraftment was faster with a Haplo donor. Importantly, the risk of NRM, death, or having a GRFS-related event was all higher in UCBT patients.

Differences in OS and LFS in favor of Haplo transplantation are the results of increased NRM with SUCBT. The difference in NRM was mainly driven by infectious complications since the incidence of acute and chronic GvHD was similar between groups. Several factors might have contributed to the excess NRM observed in the SUCBT. First, consistent with previous publication, engraftment with CB was inferior [40]. These issues could translate to an incidence of life-threatening infections. Indeed, infection accounted for 45% of deaths in the SUCBT vs. 35% in NTD-Haplo transplantation. Novel strategies for CB stem cell expansion and others facilitating engraftment kinetics equivalent to other graft sources may help to overcome this limitation [41]. Another important difference is the use of ATG, mainly in patients undergoing SUCBT. Retrospective analyses have found ATG to be associated with worse outcomes after myeloablative and reduced intensity conditioning in the setting of UCBT [42–44]; possibly due to a delayed immune recovery with ATG and increased incidence of post-transplantation lymphoproliferative disorder and infections [43, 45–48].

Finally, Haplo transplantations were performed more recently compared to SUCBT (median year of transplantation 2014 vs. 2011, *p* < 0.001), possibly accounting for lower rates of NRM in the former (20.6% vs. 48.4%, *p* < 0.001) due to improvements in supportive care. Still, one would not expect such a major difference solely on the basis of year of transplantation. Furthermore, in a multivariable analysis, adjusting for transplantation year, the benefit of Haplo was maintained.

TBF is widely used in the setting of SUCBT. Sanz et al. reported in 2012 on a single center experience of 88 patients with hematologic malignancies, who were treated with a SUCBT after conditioning with TBF-MAC [26]. Over 90% of patients engrafted at a median of 19 days. Furthermore, the 5-year cumulative incidence of NRM and relapse were 44% and 18%, respectively. Ruggeri et al. have found a similar incidence of relapse and lower NRM (33%) in acute leukemia patients treated with SUCBT and TBF-MAC [27]; outcomes were evaluated at 2 years following transplantation. The rather low relapse rates reported in these two analyses have paved the way for the increasing use of TBF-MAC for SUCBT. Indeed, our results further support the anti-leukemia effect of TBF not only in SUCBT but also with Haplo transplantations, both groups experiencing relatively low relapse rates. The effectiveness of the TBF regimen may be related to the combination of two alkylating agents, as shown in other regimens (e.g., busulfan and melphalan or carmustine [BCNU] and melphalan) [49]. More recently, our group compared TBF to a fludarabine-busulfan protocol in AML patients. Relapse rate was lower in the former, suggesting a stronger anti-leukemic effect with two alkylating agents [33]. In an additional study, the likelihood of relapse was lower with TBF compared to busulfan-cyclophosphamide, indicating that even within possible combinations of alkylating agents, thiotepa confers an additional anti-leukemia advantage [50].

Aside from donor type, additional prognostic factors were observed in our population. Low-performance status was a major predictor of relapse, decreased LFS and GRFS. Poor performance status may be a confounder of disease aggressiveness and exposure to multiple treatments. Therefore, it is difficult to determine its independent merit. Our analysis was not designed to study the importance of donor–recipient sex mismatch in the alternative donor setting. However, we found that transplantation from a female donor to male recipients was associated with an increase in NRM risk, without an apparent reduction of relapse. The results are in line with findings described by Wang and others [51–53].

The current study has several limitations. First, being a retrospective registry-based study, unknown or unmeasured factors could influence the results. However, such studies provide useful guidelines for clinical practice while waiting for randomized trials comparing specific conditionings in defined transplant settings Second, the GvHD prophylaxis strategy varied within each transplantation type. Nonetheless, both the ATG and PTCY are established options in the setting of NTD-Haplo [31, 32, 54]. Finally, a minority of patients in the SUCBT group received grafts with a total nucleated cell dose below 3 × 10^7^/kg, thereby, possibly contributing to the higher incidence of NRM in this group [55]. Yet, since only 20% grafts with less than 2.5 × 10^7^/kg, it is unlikely that changes in NRM can be entirely attributed to the cell dose.

The results of the current analysis validate the effectiveness of TBF-MAC as a potent conditioning platform allowing for graft-versus-leukemia, regardless of the type of alternative donor. While 2-year relapse risk was similar between NTD-Haplo and SUCBT in the current analysis, OS, GRFS, and NRM were superior with the former. Efforts to decrease toxicity and transplant-related mortality needs to be done to further improve outcomes. Nonetheless, a decisive conclusion that NTD-Haplo is preferable is still premature. Prospective trials comparing the two donor types are currently ongoing, and the results will help to clarify the place of the type of graft in the algorithm of donor selection. Second, UCBT safety is likely to improve with the introduction of novel technologies for stem cell expansion and better graft selection. Third, the selection of an alternative donor is mostly dependent on center preference. Currently, most institutions performing these types of transplantation are highly experienced.

## Conclusion

This retrospetive analysis suggest that TBF conditioning at a myeloablative dose enables a potent graft-versus-leukemia, regardless of the alternative donor type. Furthermore, in patients receiving TBF, survival with NTD-Haplo may be better compared to SUCBT due to decreased NRM.

## Additional file

Additional file 1: **Table S1.** HLA haploidentical transplantation strategy. **Table S2.** GvHD prophylaxis in patients receiving NTD-Haplo. **Table S3.** GvHD prophylaxis in patients receiving single umbilical cord blood transplantation. **Table S4.** Causes of Death. **Table S5.** The impact of MRC cytogenetic risk groups. (DOCX 19 kb)

## Abbreviations

ALWP: Acute Leukemia Working Party
AML: Acute myeloid leukemia
ATG: Anti-thymocyte globulin
CBUs: Cord blood units
CR: Complete remission
EBMT: European Society for Blood and Marrow Transplantation
GRFS: GvHD-free relapse-free survival
GvHD: Graft-versus-host disease
Haplo: Haploidentical stem cell transplantation
HLA: Human leukocyte antigen
HR: Hazard ratio
HSCT: Hematopoietic stem cell transplantation
KPS: Karnofsky performance status
LFS: Leukemia-free survival
MAC: Myeloablative conditioning
NRM: Non-relapse mortality
NTD: Non-T cell depleted
OS: Overall survival
PTCY: Post-transplant cyclophosphamide
RI: Relapse incidence
SUCBT: Single unit umbilical cord blood transplantation
TBF: Thiotepa–IV busulfan–fludarabine
UCBT: Unrelated umbilical cord blood transplantation
