# Supplementary material for: Haploidentical transplantation is associated with better overall survival when compared to single cord blood transplantation: an EBMT-Eurocord study of acute leukemia patients conditioned with thiotepa, busulfan, and fludarabine
Source: J Hematol Oncol. 2018 Aug 30;11:110. doi: 10.1186/s13045-018-0655-8 (PMC6117895; doi:10.1186/s13045-018-0655-8)
Supplement: Supplementary file 1 — Table S1. HLA haploidentical transplantation strategy. Table S2. GvHD prophylaxis in patients receiving NTD-Haplo. Table S3. GvHD prophylaxis in patients receiving single umbilical cord blood transplantation. Table S4. Causes of Death. Table S5. The impact of MRC cytogenetic risk groups. (DOCX 19 kb) [file 13045_2018_655_MOESM1_ESM.docx]

**Supplementary**

**Table S1: HLA haploidentical transplantation strategy**

|  | | ATG | | Total |
| --- | --- | --- | --- | --- |
|  |  | No | Yes |  |
| PTCY | No | 10 | 42 | 52 |
|  | Yes | 121 | 11 | 132 |
| Total* | | 131 | 53 | 184 |

* Data on PTCY of two patients receiving ATG was missing, therefore, these patients were not included in the table.

Human leukocyte antigen (HLA), anti-thymocyte globulin (ATG), post transplantation cyclophosphamide (PTCY)

**Table S2: GvHD prophylaxis in patients receiving NTD-Haplo**

|  | No PTCY | PTCy |
| --- | --- | --- |
| Cyclosporine | 3 | 5 |
| Mycophenolate mofetil | 0 | 3 |
| Sirolimus | 1 | 0 |
| Tacrolimus | 0 | 2 |
| Cyclosporine + Methotrexate | 10 | 2 |
| Cyclosporine + Mycophenolate mofetil | 9 | 101 |
| Methotrexate + Mycophenolate mofetil | 0 | 1 |
| Mycophenolate mofetil + Tacrolimus | 0 | 16 |
| Cyclosporine + Methotrexate + Mycophenolate mofetil | 29 | 0 |
| Other | 0 | 2 |
| Total | 52 | 132 |

Graft-versus-host disease (GvHD), non-T-cell depleted haploidentical transplantation (NTD-Haplo), post-transplantation cyclophosphamide

**Table S3: GvHD prophylaxis in patients receiving single umbilical cord blood transplantation**

|  | N | % |
| --- | --- | --- |
| Cyclosporine | 76 | 52.1 |
| Cyclosporine + Mycophenolate mofetil | 63 | 43.2 |
| Cyclosporine + Methotrexate | 4 | 2.7 |
| Other | 3 | 2.1 |
| missing | 1 |  |
| Total | 147 |  |

Graft-versus-host disease (GvHD)

**Table S4: Causes of Death**

| causes of death | **NTD-Halpo** | | **SUBCT** | |
| --- | --- | --- | --- | --- |
|  | N | % | N | % |
| Infection | 17 | 34.7% | 32 | 40.0% |
| Original disease | 13 | 26.5% | 13 | 16.3% |
| Graft-versus host disease | 10 | 20.4% | 15 | 18.8% |
| Sinusoidal obstructive sydnrome | 3 | 6.1% | 2 | 2.5% |
| idiopathic pneumonia syndrome | 3 | 6.1% | 1 | 1.3% |
| other | 2 | 4.1% | 13 | 16.3% |
| hemorrhage | 1 | 2.0% | 4 | 5.0% |
| missing | 3 |  | 0 |  |
|  | 52 |  | 80 |  |

non-T-cell depleted haploidentical stem cell transplantation (NTD-Haplo), single umbilical cord blood transplantation (SUBCT)

**Table S5: The impact of MRC cytogenetic risk groups**

|  |  | Relapse | NRM | LFS | OS | GRFS |
| --- | --- | --- | --- | --- | --- | --- |
| **2-years outcomes according to MRC cytogenetic risk group** | good | 10.3%[2.5-24.7] | 31.2%[15.3-48.6] | 58.4%[40.4-76.4] | 61.6%[43.7-79.4] | 53.3%[34.8-71.9] |
|  | intermediate | 12%[6.9-18.8] | 32.8%[24.4-41.4] | 55.2%[46.1-64.3] | 59.4%[50.5-68.4] | 47.1%[37.9-56.3] |
|  | poor | 27.4%[13.4-43.6] | 28.9%[13.8-45.9] | 43.7%[25.8-61.5] | 49.2%[31-67.3] | 40.4%[22.9-57.9] |
|  | P value | 0.047 | 0.88 | 0.42 | 0.54 | 0.56 |
| **2-years outcomes in patients with intermediate risk cytogenetics** | NTD-Haplo (n=67) | 13.7%[6.3-24] | 19.1%[10.4-29.9] | 67.2%[55.2-79.2] | 75.7%[64.9-86.5] | 58.6%[45.9-71.2] |
|  | SUCBT (n=60) | 10.2%[4.1-19.7] | 47.2%[33.5-59.6] | 42.6%[29.6-55.5] | 42.5%[29.5-55.4] | 35.4%[22.9-48] |
|  | P value | 0.962 | 0.002 | 0.003 | 0.000 | 0.002 |

Medical Research Council (MRC), non-T-cell depleted haploidentical stem cell transplantation (NTD-Haplo), single umbilical cord blood transplantation (SUBCT), non-relapse mortality (NRM), leukemia-free survival (LFS), overall survival (OS), graft-versus-host-free relapse-free survival (GRFS)
